# Tabes Dorsalis in a Patient Presenting With Right Lower Extremity Paresthesia and Cervical Spine Pain

**DOI:** 10.7759/cureus.14011

**Published:** 2021-03-20

**Authors:** Kellen T Creech, Komal M Patel, Umar Chaudhry

**Affiliations:** 1 Internal Medicine, Dr. Kiran C. Patel College of Osteopathic Medicine, Nova Southeastern University, Fort Lauderdale, USA; 2 Family Medicine, Dr. Kiran C. Patel College of Osteopathic Medicine, Nova Southeastern University, Fort Lauderdale, USA; 3 Internal Medicine, HCA Westside-Northwest, Plantation, USA

**Keywords:** tabes dorsalis, neurosyphilis, syphilis

## Abstract

Syphilitic myelitis, also known as tabes dorsalis, is a disease affecting the posterior columns of the spinal cord and dorsal roots and presents as sensory ataxia and neuropathic pain and less commonly as paresthesia and gastrointestinal disturbance. Tabes dorsalis is the clinical manifestation of a previous infection with syphilis, and the average latency period from initial infection to presentation of symptoms is approximately 25 years. This is a rarely encountered manifestation of syphilis since the widespread usage of antibiotics. Penicillin G is the mainstay therapy of neurosyphilis and has been shown to improve and resolve spinal cord lesions associated with tertiary syphilis. We present a case of tabes dorsalis in a 56-year-old female with a history of extensive autoimmune disease who initially presented with neck pain and numbness of the right lower extremity. The unique nature of this case lies in the patient’s clinical course, as her symptoms were initially attributed to her history of autoimmune disease. A reactive CSF-VDRL (cerebrospinal fluid Venereal Disease Research Laboratory) test and MRI findings led clinicians to suspect neurosyphilis and begin penicillin G. The patient began to show significant clinical improvement after penicillin G therapy was begun and was discharged to a rehabilitation facility to continue antibiotics and begin aggressive physical therapy.

## Introduction

*Treponema pallidum*, a gram-negative spirochete, is the causative organism of the sexually transmitted infection known as syphilis [1]. Worldwide, there were an estimated number of 19.9 million cases of syphilis, with 6.8 million new cases reported in individuals between 15 and 49 years of age, as reported by the World Health Organization [1]. The United States experienced a mini-epidemic of syphilis in the 1980s and early 1990s, as the number of cases peaked at 20.3 cases per 100,000 people [1]. Although cases of syphilis decreased over the next decade, cases of primary and secondary syphilis in the United States have steadily increased since 2001, especially in women [1]. Colloquially known as the “great imitator,” clinical manifestations of syphilis widely vary and depend upon the stage of disease (primary, secondary, and tertiary). In the pre-antibiotic era, tertiary syphilis displayed neurological symptoms in 25-35% of patients [2]. Tabes dorsalis, a disease of the posterior columns of the spinal cord and dorsal roots, developed in around 10% of patients after a latency period of approximately 25 years [2]. Since the widespread usage of antibiotics, syphilitic myelitis, also known as tabes dorsalis, is uncommonly seen, although a history of HIV infection put patients co-infected with syphilis at an increased risk [2]. This case was selected due to an unusual presentation of tabes dorsalis in a patient with multiple autoimmune conditions. This case highlights the importance of taking a thorough history, where it was revealed that this patient had an untreated sexually transmitted infection many years ago, which led the clinician to suspect and diagnose neurosyphilis.

## Case presentation

**Figure 1:**
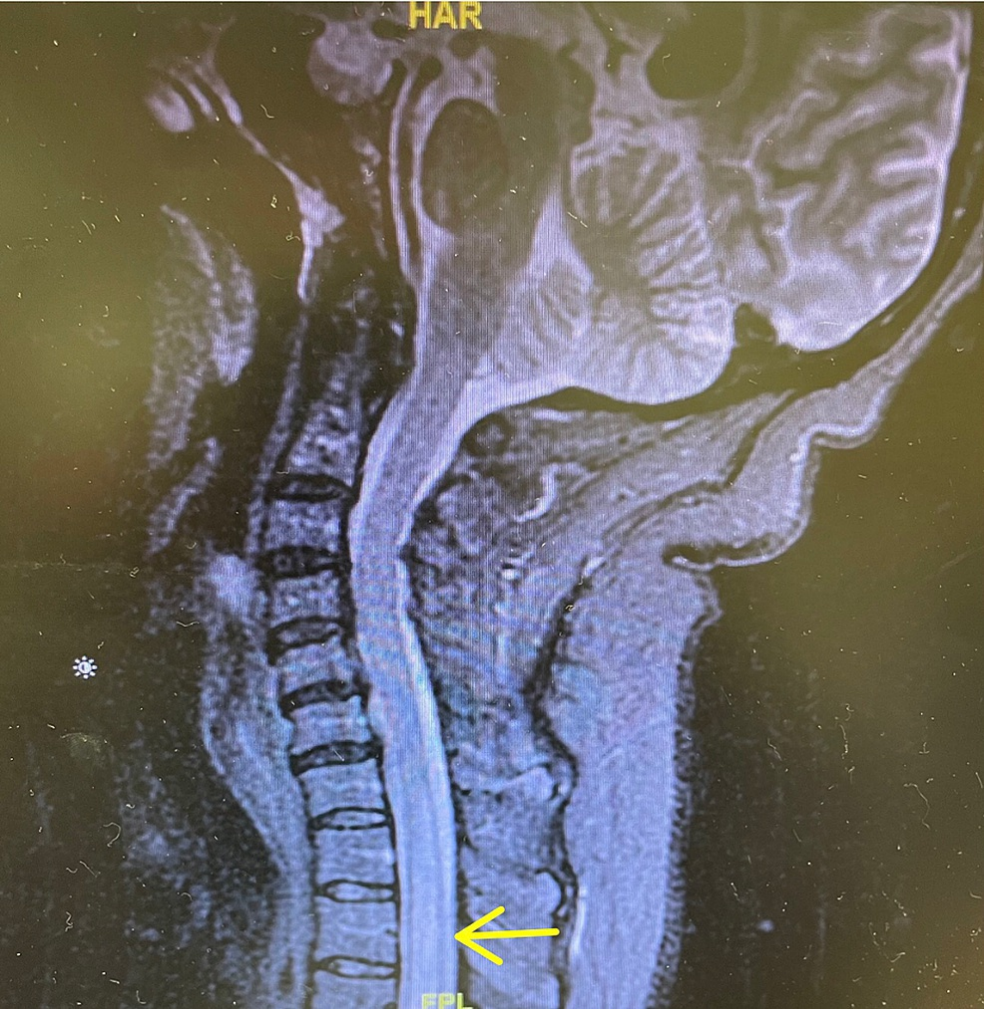
T2-weighted cervical spine MRI showing hyper-intense intramedullary lesion beginning at the level of C7 (yellow arrow).

**Figure 2:**
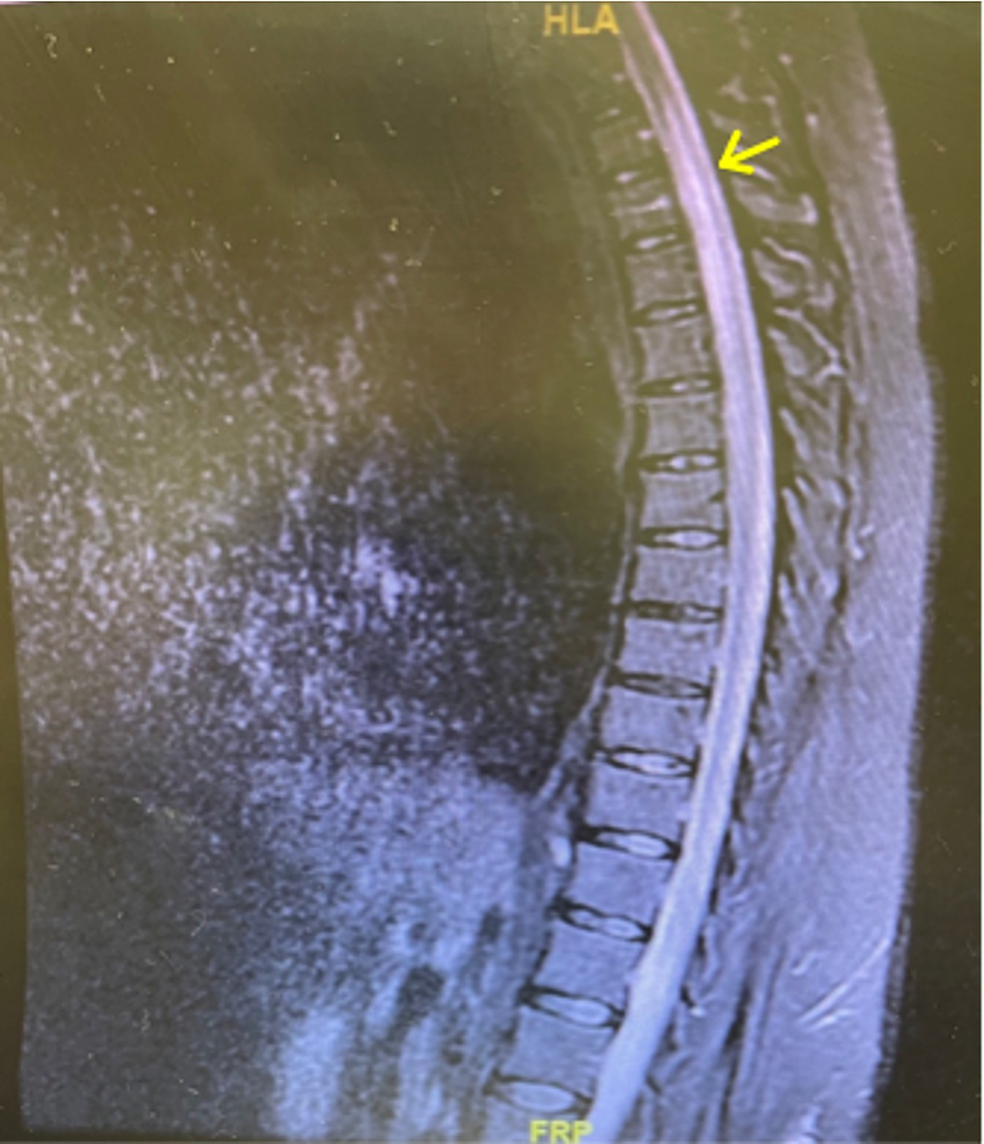
T2-weighted thoracic spine MRI showing hyper-intense intramedullary lesion from the level of C7 to T7 (yellow arrow).

**Figure 3:**
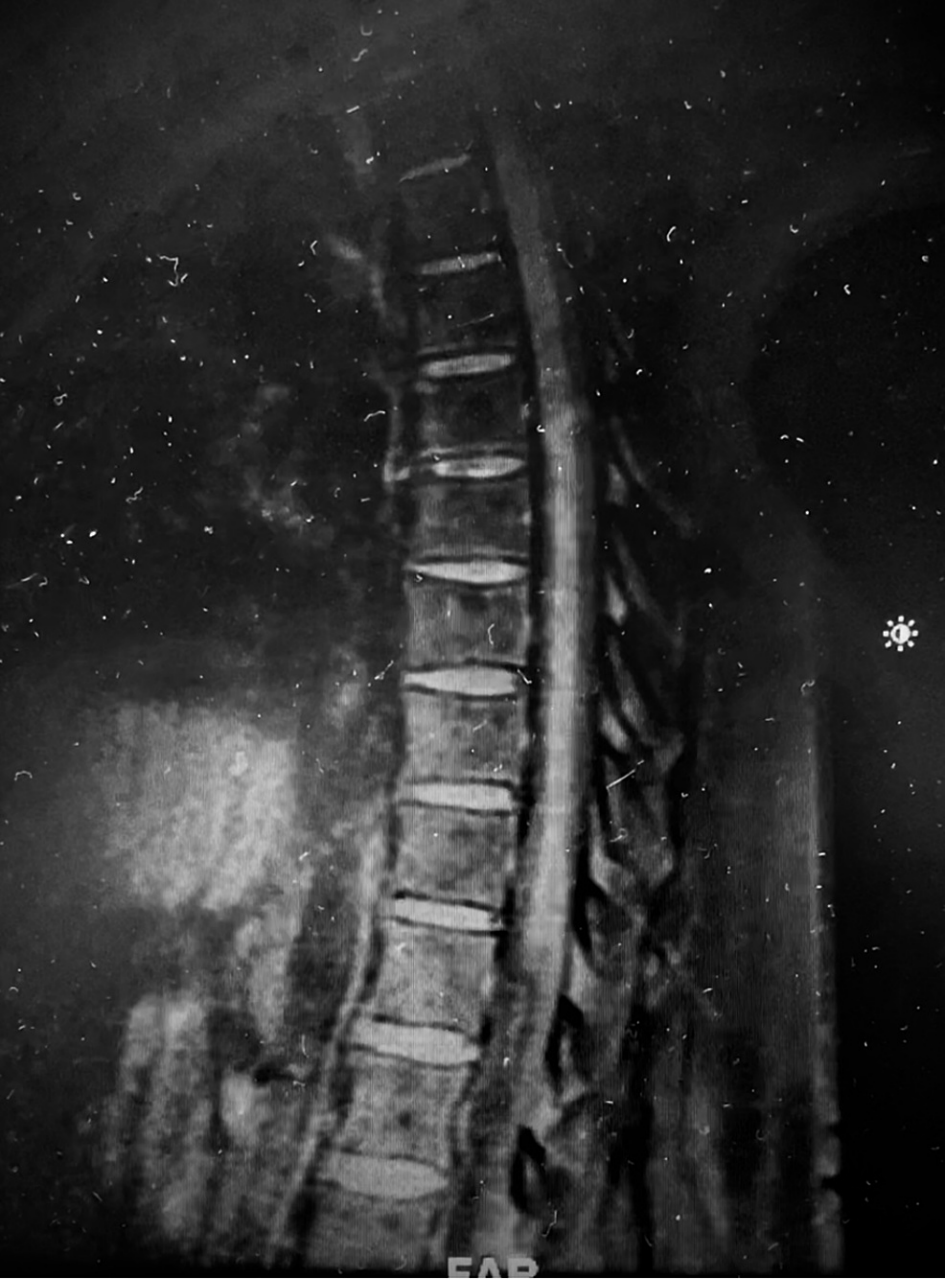
T1-weighted thoracic spine MRI pre-contrast (gadolinium).

**Figure 4:**
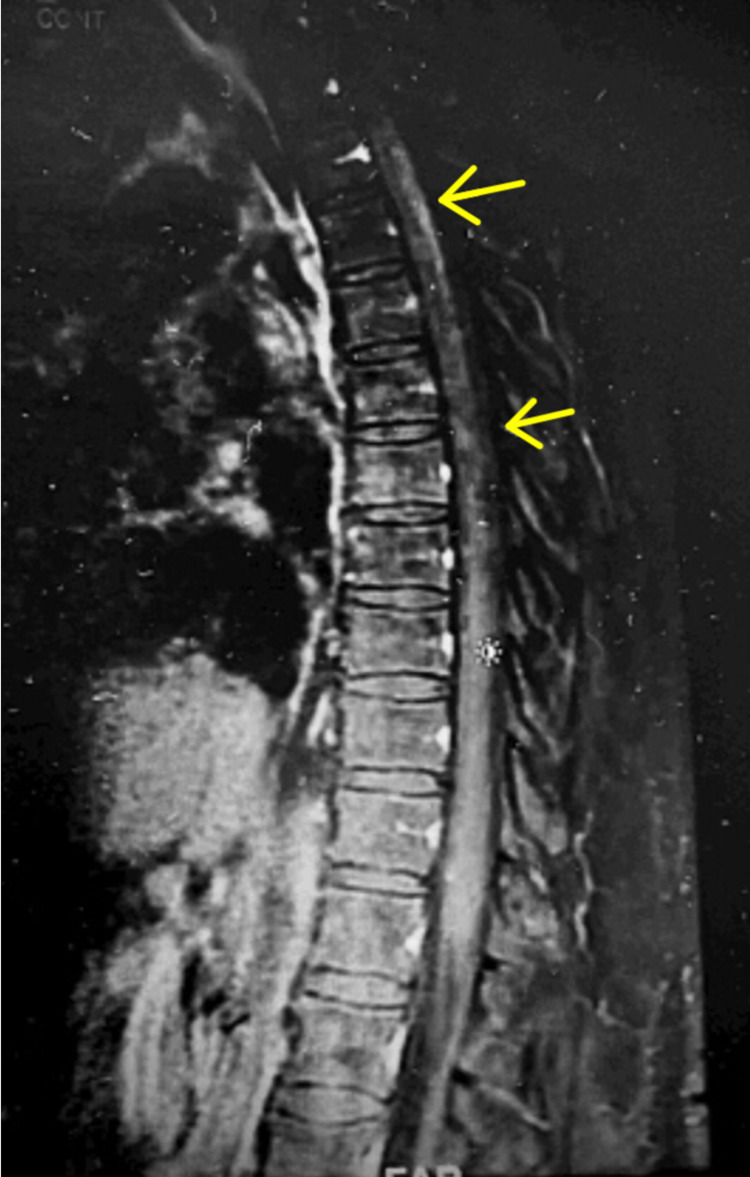
T1-weighted thoracic spine MRI post-contrast (arrows denoting “candle-guttering” appearance, characteristic of syphilitic myelitis).

A 56-year-old African American female with a medical history significant for systemic lupus erythematous, Sjögren’s syndrome, rheumatoid arthritis, fibromyalgia, non-insulin-dependent diabetes mellitus, dyslipidemia, and obesity presented to the emergency department with the complaints of right foot ascending paresthesia and posterior-inferior cervical spine pain that began on the same day of admission. She stated that the symptoms began abruptly and involved her right lower extremity and then extended to include her left lower extremity. Physical examination was significant for sensory deficit level T5 and below left greater than right side, mild bilateral leg weakness 4/5, reflexes equal and bilateral, and cranial nerves II-XII intact, and the rest of the examination was unremarkable. Her symptoms gradually continued during her hospitalization until the numbness encompassed her entire body from her mid-chest region to her bilateral lower extremities. She denied experiencing any accompanying headaches, dizziness, fever, chills, nausea, vomiting, rhinorrhea, sore throat, cough, chest pain, dyspnea, abdominal pain, diarrhea, flank pain, dysuria, polyuria, hematuria, or myalgias. Our patient mentioned that she was a native of a country in the Caribbean, but she moved to the United States in the late 1970s. She denies any recent travel, sick contacts, recent antibiotics use, animal exposure, or history of multi-drug resistant infections. Magnetic resonance imaging (MRI) of the cervical and thoracic spine revealed lesions consistent with myelitis with extensive involvement from C7 to T7 (Figures 1, 2). She was initially treated with a round of corticosteroids followed by intravenous immunoglobulin (IVIG) therapy. She reported experiencing persistent paresthesia from the level of the xiphoid process down that was unchanged since admission. Infectious disease and neurology services were consulted, and the patient reported experiencing a cold-like illness three weeks prior to admission and was on an unknown antibiotic at that time. Chest X-ray was significant for increased lung markings and mild pulmonary congestion without acute airspace disease. Computed tomography angiogram demonstrated no evidence of pulmonary embolism, aortic aneurysm, or aortic dissection. T2-weighted MRI images of the cervical/thoracic spine demonstrated hyper-intensities within the lower cervical and upper thoracic cord, extending from the levels of C7 to T7. There was also notable enhancement through this region on post-contrast T1-weighted MRI images (Figures 3, 4).

MRI of the brain showed no acute intracranial process. Blood cultures repeated twice were negative for growth of organisms. Human immunodeficiency virus (HIV) testing was nonreactive. Further discussion with our patient revealed a history of an untreated sexually transmitted infection years ago as a young woman in the Caribbean. This led clinicians to consider other causes, and a lumbar puncture was ordered. Cerebrospinal fluid (CSF) cytology showed no organisms on gram stain and no growth. CSF Cryptococcus antigen (Ag) was negative. CSF-VDRL (cerebrospinal fluid Venereal Disease Research Laboratory) test was positive. Subsequent to the reactive CSF-VDRL test, infectious disease service initiated penicillin G (4 million units intravenous [IV] every four hours) therapy for a total of 14 days. After receiving IV penicillin G, she reported significant clinical improvement. Our patient was stabilized and transferred to acute rehab to continue IV antibiotics and begin aggressive physical therapy.

## Discussion

Tabes dorsalis is a rarely encountered neurological manifestation of syphilis in the antibiotic era. Norwegian physicians in the late 19th century began to further understand syphilis by describing the unique evolution of this infection in 1400 patients with primary and secondary syphilis [1]. Additional information was compiled on the late manifestations of syphilis through pathological findings on the autopsies of 382 adults between 1917 and 1941 [1]. Considering that the latency period between the initial infection and manifestations of tabes dorsalis is approximately 25 years, how do patients allow for these infections to go untreated for so long? This is explained by the manifestations of primary syphilis and the widely varying symptoms of secondary and tertiary syphilis. Primary syphilis is initially manifested by a painless papule referred to as a “chancre” [3]. This lesion tends to ulcerate and commonly appears as a 1 to 2 centimeter ulcer with an elevated border with mild-to-moderate regional lymphadenopathy [3]. These lesions heal spontaneously within three to six weeks even without treatment administered [3]. If syphilis continues to go untreated, 1 to 30 years later, 25% to 40% of patients may present with a variety of symptoms constituting late or tertiary syphilis [2]. The most common manifestations of late manifestations include aortitis, granulomatous nodular lesions, general paresis, and tabes dorsalis [2]. In specifically discussing tabes dorsalis, the most common symptoms are sensory ataxia and lancinating pains, but one of the less common symptoms is paresthesia (numbness or a “pins and needles” sensation), which our patient exhibited [2]. This case adds to current scientific literature because although the number of cases of primary and secondary syphilis has been increasing, the reported prevalence of neurosyphilis among reported cases of syphilis in the United States from 2009 to 2015 was reported at 0.84% due to the widespread usage of antibiotics [4]. Lumbar puncture should be considered in a patient presenting with neurologic, otolgic, or ocular symptoms that could be attributed to syphilis but have an unknown history of prior infection. In an evaluation of CSF in a patient with suspected tertiary syphilis, a reactive CSF-VDRL test is specific for syphilis and can establish the diagnosis of neurosyphilis [5]. However, the sensitivity of this test is poor and can be negative in as many as 70% of patients with neurosyphilis [5]. In a study of 40 patients with presumed neurosyphilis, only 30% of patients exhibited a reactive CSF-VDRL test [5]. Therefore, the absence of a highly sensitive and specific test for neurosyphilis complicates the diagnostic process for patients suspected of having this disease [5]. MRI imaging of the spine is also important in the diagnosis of syphilitic myelitis. Hyperintense lesions appreciated on T2-weighted images are often characteristic in cases of syphilitic myelitis [6]. Also, T1-weighted MRI images that show enhancement after gadolinium administration are what is known as the “flip-flop sign” or “candle-guttering” appearance, characteristic of syphilitic myelitis [6]. These lesions are due to spinal cord ischemia and meningeal inflammation and have been shown to improve and resolve upon penicillin G therapy [6]. The lesions found on the MRI alone do not provide enough substantial evidence to consider penicillin G therapy because these lesions can resemble other pathological causes of myelitis. In this case, the CSF-VDRL test in conjunction with the MRI findings confirmed the diagnosis of syphilitic myelitis and led to initiating penicillin G therapy. IV penicillin G (3-4 million units IV every four hours or 18-24 million units per day by continuous infusion) for 10 to 14 days is the mainstay therapy for syphilis infection, and our patient began to show significant clinical improvement after penicillin G treatment was initiated [7]. This case also highlights the significance of a proper social history to screen for a past history of sexually transmitted infections in the setting of a patient presenting with a constellation of vague neurological symptoms. Early in the case, the patient’s symptoms were assumed to be due to her history of autoimmune disease. However, our patient did not improve while on steroids, and a more thorough review of her social history raised the suspicion for neurosyphilis due to an untreated sexual infection many years ago.

## Conclusions

Tabes dorsalis is the neurological manifestation of a previous infection with syphilis after an average latency period of 25 years. Affecting the posterior columns of the spinal cord and dorsal roots, this disease commonly presents as ataxia and neuropathic pain and less commonly as paresthesia. Although physical examination findings can vary, the presence of characteristic findings such as the “flip-flop” sign or “candle-guttering” appearance on enhanced T1-weighted MRI images, hyperintense lesions on T2-weighted MRI images, and a positive CSF-VDRL test can establish the diagnosis of neurosyphilis. Upon diagnosis, penicillin G therapy is the mainstay therapy, and lesions have been shown to improve and resolve with adequate antibiotic coverage. Although the occurrence of tabes dorsalis is rarely seen today since the widespread usage of antibiotics, this case reminds of the importance of a wide differential and thorough social history in patients immigrating from other countries and complaining of vague neurological symptoms.

## References

[1] Hicks CB, Clement M, Marrazzo Marrazzo, J. J. (2020). Syphilis: epidemiology, pathophysiology, and clinical manifestations in patients without HIV. UpToDate.

[2] Marra CM (2015). Neurosyphilis. Continuum (Minneap Minn).

[3] O’Byrne P, MacPherson P (2019). Syphilis. BMJ.

[4] de Voux A, Kidd S, Torrone EA (2018). Reported cases of neurosyphilis among early syphilis cases—United States, 2009 to 2015. Sex Transm Dis.

[5] Hicks CB, Clement M (2021). Syphilis: screening and diagnostic testing. UpToDate.

[6] Sun L, Zheng N, Yang Y, Zhang HN (2018). Syphilitic meningomyelitis presenting with visceral crisis: a case report. Medicine (Baltimore).

[7] Hicks CB, Clement M. (2021). Syphilis: treatment and monitoring. UpToDate.

